# The Effect of Specific Immunotherapy on Natural Killer T cells in Peripheral Blood of House Dust Mite-Sensitized Children with Asthma

**DOI:** 10.1155/2012/148262

**Published:** 2012-09-13

**Authors:** Lu Yan-ming, Cao Lan-fang, Li Chen, Li Ya-qin, Chen Wei, Zhang Wen-ming

**Affiliations:** Department of Pediatrics, Renji Hospital, Shanghai Jiaotong University School of Medicine, Shanghai 200001, China

## Abstract

To investigate the effects of specific immunotherapy on the NKT cells in peripheral blood and the ability of NKT cells to proliferate in response to alpha-galactosylceramide (alpha-GalCer) in house-dust-mite- (HDM-) sensitized asthma children, peripheral blood mononuclear cells were isolated from 42 asthmatic children, of whom 24 were on specific immunotherapy (SIT) for more than a year and 20 were healthy. Compared with control group, the ratio of peripheral blood NKT and CD4^+^NKT cells was significantly decreased (*P* < 0.01) and was elevated in SIT asthma group (*P* < 0.05), respectively, but it was still less than the normal control group (*P* < 0.01). The level of IL-4 in serum secreted by NKT cells in asthma group was significantly higher than that of control group (*P* < 0.01), particularly apparent after 72 hours. The level of IL-4 in SIT group decreased significantly (*P* < 0.01). The level of IL-10 in serum secreted by NKT cells in asthma group was decreased significantly than that of the control group (*P* < 0.01) especially in 48 hours, while that of SIT group was increased significantly (*P* < 0.01). These results suggest that the pathogenesis of asthma may be related to the ratio and dysfunction of NKT and CD4^+^NKT cells.

## 1. Background

Natural Killer (NKT) T cells express not only NK1.1 antigen (CD161 in human, NKR-PIC in mouse), a unique marker of NK cells, but also the T-cell receptor (TCR). The NKT cells are divided into two types: type I NKT cells and type II NKT cells. Type I NKT cells express constant TCR *α* chain, which are also named as invariant natural killer T cells (iNKT), constituting about 70–80% of all NKT cells. T-cell receptor of Human iNKT cells consists of constant V*α*24-J*α*18 chain and V*β*11 chain, while rodents consist of V*α*14-J*α*18 and V*β*8.2, V*β*7 or V*β*2 chain, which mainly recognize *α*-GalCer presented by CD1d. Human NKT cells can be further divided into three subtypes: CD4^+^NKT cells, CD4^−^CD8^−^NKT (DN NKT) cells, and CD8^+^NKT cells [1].

iNKT comprises of T lymphocyte lineage which is extremely restricted to T-cell antigen receptor with limited junctional diversity (V*α*24-J*α*18 in human and V*α*14-J*α*18 in mouse). One of the features that distinguishes iNKT from conventional T cells is their capacity to rapidly and in some cases simultaneously produce large amounts of both T helper (Th) 1 proinflammatory (promoting cellular immune responses) and Th2-type anti-inflammatory (promoting certain antibody responses) cytokines upon triggering, thereby mediating the regulation of a variety of immune responses, including antitumor immune responses [2].

It has been seen that the specific role of NKT cells in the development of asthma is complex. Although the development of Th2-driven responses to allergens is an important factor in the development of asthma, iNKT cells may provide a lung-specific mechanism that allows asthma to develop in allergic individuals. In this process, iNKT cells may in some way process Th2 cells to induce AHR or may enhance Th2 cell development [3]. Investigations on occurrence, development, and treatment options of asthma with various means of immunomodulation have gradually attracted the attention of clinicians. The WHO explicitly pointed out that the standard specific immunotherapy (SIT) is the only way to change the natural processes of allergic diseases that occur due to the treatment. The SIT can improve the quality of life: relieving the symptoms of patients with asthma, improving lung function, and reducing the use of emergency drugs. However, sometimes SIT cannot completely control asthma, and some patients still require long-term conventional drug treatment after SIT treatment. In addition, SIT can also cause systemic and/or local side effects, or even fatal anaphylactic shock. The efficacy and safety of SIT in the treatment of allergic asthma has yet to be solved, one reason could be nonunderstanding of the exact mechanism of SIT in asthma treatment. Therefore, to explore further the mechanism of SIT in asthma treatment has great significance in improving its efficacy and safety and further elucidating the pathogenesis of asthma.

Using TCR V*α*24^+^/TCR V*β*11^+^ double-positive cells as NKT cells, we observed changes in the number of NKT cells and CD4^+^NKT cells TCR V*α*24/TCR V*β*11 cells by immunofluorescent labeling and flow cytometry method in children with asthma, children with asthma treated with standard SIT for more than 1 year, and normal children. Following which, we observed expression pattern change of cytokines (IL-4, IL-10, IFN-*γ*) produced by NKT cells after stimulating peripheral blood mononuclear cells with *α*-GalCer, specific agonist of NKT cells. *α*-GalCer is often used to test and isolate and activate NKT cell specifically working as specific activator of NKT cell. They were stimulated repeatedly by *α*-GalCer *in vitro* which can reflect the secretion of the NKT cell indirectly. Accordingly, the theoretical and experimental basis was provided for exploring the regulatory role of NKT cells in the pathogenesis of asthma in children and specific immunotherapy.

## 2. Materials and Methods

All 66 cases were children (39 male and 27 female) with asthma diagnosed by Pediatric clinic of our hospital from February 2009 to March 2012. All the children were aged 8.57 ± 3.01 years (range: 5 to 16 years) and met the diagnosis and prevention guidelines for children with bronchial asthma developed by the Respiratory Group of Pediatrics Branch, Chinese Medical Association [4], with or without allergic rhinitis. The medical history of the cases was with dust mite allergy through history, allergen skin pricks test (SPT), and the *in vitro* serum allergen-specific IgE determination. Other inclusion criteria were presence of at least one mite allergy (household dust mites, dust mites, tropical mites) and the range of SPT wheal reaction ≥ ++ compared with histamine. Patients who had autoimmune diseases except allergic diseases, mental disorders, heart, liver and kidney disorders were excluded. Caretakers for all the patients signed informed consent. 

### 2.1. Asthma Group

Patients without the implementation of SIT-, 26 cases were classified as mild intermittent, 10 cases as mild persistent, and 6 cases as moderate persistent in accordance with the asthma severity. The whole group underwent careful history taking, physical examination, and even a chest X-ray to exclude pulmonary infection when necessary.

### 2.2. SIT Group

This group included 24 children with asthma (12 males and 12 females) with average age 10.8 ± 3.40 years (range: 5–16 years), application of standard allergen preparations for adustmite (Alutard, ALK-Abello Company, Denmark) for at least 1 year.

### 2.3. Control Group

Twenty healthy children (12 males and 8 females) with mean age 8.95 ± 3.81  years had no recent infection or a history of allergic disease.

### 2.4. Main Reagents**  **and Instruments

Lymphocyte separation medium Ficoll-Hypaque (Shanghai Second Reagent Factory); erythrocyte lysis buffer (Cogent Bio); anti-human CD4-APC (eBioscience); TCRV*α*24-PE and TCRV*β*11-FITC (Backmancoulter, U.S.); alpha-Galcer (Enzo Company, USA); RPMI-1640 medium, fetal bovine serum (Gibco, USA); ELISA Kit for IL-4, IL-10, IFN-gamma (RD Corporation, USA); flow cytometer BDFACS Caliber flow (BD, USA).

### 2.5. Experimental Methods

#### 2.5.1. Peripheral Blood Mononuclear Cells (PBMNCs) Separation

Peripheral blood about 4-5 mL was collected aseptically in children with asthma and those who were normal. Anticoagulation was done with heparin, cascaded onto the lymphocyte separation solution after PBS-fold diluted, centrifuged at 2000 *r*/min for 20 min, *r* = 15.5 cm. The albuginea cell layer was collected and washed with PBS and resuspended in RPMI-1640 medium which was then counted for the future use.

#### 2.5.2. NKT Cell Detection

100 uL of peripheral blood was freshly collected aseptically from children in asthma and normal group into flow cytometry tubes; added 10 *μ*L of CD4-APC antibody, TCRV*α*24-PE antibody, and TCRV*β*11-FITC antibody; added 1 mL erythrocyte lysis solution after mixture and incubation. After full lysis, excluded supernatant after centrifugation for 3 minutes at 2500 *r*/min, *r* = 13.8 cm was washed with 2 mL PBS, then NKT cells and CD4^+^NKT cells were detected with flow cytometry. The anti-human TCR V24V-11-monoclonal antibody was the specific marker of NKT cells.

#### 2.5.3. Amplification and Activation of Nkt Cells

Mononuclear cells (5 × 10^5^) collected from peripheral blood were cultured in RPMI-1640 medium containing 10% fetal bovine serum with *α*-Galcer (final concentration: 100 ng/mL) in 25 cm^2^ culture flask bottle, placed into 5% CO_2_, at 37°C incubator. The culture supernatant was collected at 24 h, 48 h, and 72 h, respectively,

**Detection of the Level of IL-4, IFN-*γ*, IL-10 in the Culture Supernatant with ELISA**

It was measured according to ELISA kit instruction (RD Company, USA).

#### 2.5.4. Clinical Data Collection

The allergic indicators total IgE and ECP of children with asthma were detected after collecting the blood. 

#### 2.5.5. Statistic Analysis

The result for each group was expressed as $\overset{-}{x}$ ± *s* (mean ± sd), analyzed with statistical software SPSS13.0. Complete random control variance analysis was applied for the mean comparison among multiple groups. Comparison between two groups was done with the Mann-Whitney *U* test. Correlation analysis of two-variable was done with linear correlation analysis method. *P* < 0.05 was considered to be statistically significant.

## 3. Results

The baseline characteristics of the patients have been reported in the Table 1. There were no significant difference, in demographics and severity of the disease between asthma group and the SIT group. 

### 3.1. The Ratio of Peripheral Blood NKT and CD4^+^NKT Cells in Asthma Patients and Control Group

Compared with normal control group, the rate of NKT and CD4^+^NKT cells significantly decreased in children with asthma (*P* < 0.01). The ratio of NKT cells and CD4^+^NKT cells in SIT group was higher than that in asthma group (*P* < 0.05), but still lower than that in the normal control group (*P* < 0.01, Table 2).

### 3.2. The Ratio of Peripheral Blood NKT and CD4^+^NKT Cells in Asthma Patients according to Asthma Severity

In the group of children with asthma, the ratio of peripheral blood NKT cells and CD4^+^NKT cells of moderate persistent patients was slightly lower than that of mild intermittent and that of mild persistent, but there was no significant difference in the ratio of peripheral blood NKT cells and CD4^+^NKT cells among the three groups (*P* > 0.05, Table 3).

### 3.3. Correlation Analysis

There was no significant correlation among NKT cells and total IgE and ECP (*r* = −0.022, *P* > 0.05, *r* = 0.063, *P* > 0.05), and there was no significant correlation among NKT cells and total IgE and ECP as well (*r* = 0.022, *P* > 0.05, *r* = 0.107, *P* > 0.05).

### 3.4. The Level of IFN-*γ*, IL-4, and IL-10 Expression after NKT Cells Proliferation in Asthma Patients and Control

Level of IL-4 secreted by NKT cells after proliferation activation in children with asthma group was significantly higher than normal control group (*P* < 0.01), particularly at 72 h; compared with asthma patients. The IL-4 secretion decreased significantly (*P* < 0.01) in SIT group and it showed no significant difference between normal group and SIT group (*P* > 0.05) compared with normal control group. The secretion of IL-10 by NKT cell in asthma group patients decreased (*P* < 0.01), especially at 48 h, while IL-10 in SIT group increased significantly compared with asthma patients (*P* < 0.01), and there was no significant difference in IL-10 secretion between normal control group and SIT group (*P* > 0.05). It showed no significant difference in the IFN-*γ* secretion by NKT cells between asthma group and the SIT group (*P* > 0.05). Further, there was no significant correlation between NKT number and improvement of disease (Table 4).

## 4. Discussion

Specific immunotherapy is currently the only treatment method to intervene in allergic disease through the pathophysiological mechanisms. Therefore, it is widely used in clinical treatment of type I allergic diseases including bronchial asthma, mediated by IgE. The SIT treatment mechanism is complex, and most researchers are mainly focused on the role of the T cells followed by cytokine changes. The NKT cells are newly discovered group of special types of immune cells, after the activation, which can rapidly produce a large number of cytokines with immune-modulatory effects; including Th1-type cytokines (IFN-*γ* and TNF) and Th2-type cytokines (IL-4, IL-13), therefore, influencing Th1/Th2 immune balance. The production of cytokines in NKT is significantly faster than conventional T cells. Further, in the SIT group, children were assessed by the parents subjectively: in 11 (45.8%) children significant improvement was reported by the families, 8 children (33.3%) showed moderate improvement, 4 (16.7%) indicated a slight improvement, and 1 patient (4.2%) had no change in the condition.

The NKT cells in human peripheral blood are very few, only about 0.04%–0.2% [5]. Therefore, clinical research on NKT cells has been limited for a long time. However, NKT cells *in vitro* with repeated stimulation by *α*-GalCer will rapidly proliferate and can also show increased secretion of cytokines. The establishment of *in vitro* amplification of NKT cells may be able to lay a foundation for further clinical studies. The *α*-GalCer, is a kind of glycosphingolipid (GSL) extracted from the sponge, often used for specific identification, isolation, and activation of NKT cells. According to a recent study, *in vivo* or *in vitro* experiments, demonstrate that the extremely small amount of *α*-GalCer will be able to involve in the binding of the NKT cells CDld, which prompts amplification and activation of NKT cells, and consequently secrete large amounts of cytokines rapidly [6]. The KRN7000 is a new *α*-GalCer reagent, and it is also the most effective *α*-GalCer ligand for activation of NKT cells [7]. This study made the anti-human TCR V*α*24V*β*11 monoclonal antibody a specific marker of NKT cells, nucleated cells were collected using the erythrocyte lysis liquid to obtain a sufficient number of cells, and the use of flow cytometry improved the sensitivity of the NKT cells detection.

The study showed that the ratio of either NKT cells or CD4^+^NKT cells in children with asthma was significantly lower than the control group (*P* < 0.01), while the ratio of peripheral blood NKT cells and CD4^+^NKT cells showed no significant difference among children with different severity of asthma (*P* > 0.05), which may be associated with the small number of cases in this study. After SIT for more than one year, the ratio of NKT cells and CD4^+^NKT cells has increased, but it was still lower than the normal control group (*P* < 0.01). These results suggest that NKT cells and CD4^+^NKT cells may be involved in the pathogenesis of asthma. In addition, the results of this study showed no significant correlation between the ratio of NKT cells and CD4^+^NKT cells and total IgE and ECP, the common indicators of asthma (*P* > 0.05), similar to research by Ikegami et al. [8]. Thus, we have speculated that NKT cells may migrate and aggregate to the sites of inflammation. Asthma patients had significant mucosal inflammation in respiratory tract, therefore, NKT cells migrate and aggregate from peripheral blood gradually to the sites of inflammation of the respiratory mucosa to play its role in immune regulation, and ratio of NKT cells and CD4^+^NKT cells in peripheral blood further reduce, which is consistent to Akbari et al. study [9]. After SIT treatment, the migration and aggregation of NKT cells have been suppressed, so the ratio of NKT cells and CD4^+^NKT cells in the peripheral blood also increased.

Although the number of peripheral blood NKT cells is small, NKT cells can rapidly proliferate and show good immune response after repeated stimulation with *α*-GalCer *in vitro*. The NKT cells constitutively exist in the mRNA the Th2-type cytokines such as IL-4, IL-10, and Th1-type cytokines IFN-*γ*. Therefore, NKT cells can quickly generate two types of cytokines after activation, which make NKT cells unique regulatory T cells [10]. A large number of cytokines secreted by activated NKT cells play a promoting or inhibitory role in the occurrence or development of inflammatory response of asthma. After proliferation and activation of NKT cells *in vitro* by *α*-GalCer (KRN7000), the result showed that the IL-4 secreted by NKT cells increased significantly in asthma (*P* < 0.01) than normal control group, particularly at 72 hours; while the IL-4 in SIT group was significantly decreased compared with asthma group (*P* < 0.01) and there was no significant difference between normal control group and SIT group (*P* > 0.05). There was no significant difference between the asthma group and the SIT group in the secretion of IFN-*γ* by peripheral blood NKT cells (*P* > 0.05). Although previous studies suggested that successful SIT treatment is often accompanied with an increased IFN-*γ*, there are a growing number of research reports indicating that IFN-*γ* secreted by CD8^+^ T cells will further aggravate asthma disease severity, therefore, it has been questioned that IFN-*γ* can improve Th2-dominated inflammatory response in patients with asthma [11]. Our experimental studies suggest that change on the function of NKT cells in children with asthma may change the patient immune response to Th2-induced humoral immune, thus inhibiting Thl response. SIT treatment can correct the immune bias, which was expressed mainly as reduced Th2 cytokines IL-4 secreted by NKT cells, not an increase in IFN-*γ*. In addition, IL-10 secreted by NKT cell in the asthma group was significantly decreased compared with the normal control group (*P* < 0.01), especially at 48 hours, and the IL-10 of SIT group significantly increased compared with asthma patients (*P* < 0.01). There was no significant difference between control group and SIT group (*P* > 0.05). This suggests that, during SIT, repeated stimulation by allergens could induce IL-10 secretion in NKT cells. With the OVA-sensitized BALB/c mouse model, Vissers et al. found that, the relief of airway hyperresponsiveness following the reduction of EOS after immunotherapy was inhibited through knockout of IL-10 receptor, which corresponded to the decrease of specific IgE and Th2-type cytokine [12]. Therefore, increased level of IL-10 after SIT played a powerful role in induction of immune tolerance.

## 5. Conclusion

In summary, this study indicated that there was a reduction in number of peripheral blood NKT cells and CD4^+^NKT cells in patients with asthma, as well as change of cell function. Human NKT cells can produce cytokines IL-4 and IL-10, which promote Th2 cell generation and play an important role in the pathogenesis of asthma. Specific immunotherapy can correct the bias of the NKT cells number and their functions in the patients with asthma. However, due to the small size of sample, we did not compare the patients with asthma after SIT with those before SIT. We will further improve our research in order to draw more definitive conclusions in the future.

## Figures and Tables

**Table 1:** Baseline characteristics of the patients.

	Age	Gender (Male/Female)	T IgE (IU/mL)	Ecp (ug/L)
Control	8.95 ± 3.81	12/8	461.45 ± 476.33	28.37 ± 25.34
Asthma	8.57 ± 3.01	27/15	518.56 ± 426.35	43.22 ± 30.97
SIT	10.08 ± 3.40	12/12		

**Table 2:** The ratio of peripheral blood NKT and CD4^+^NKT cells in asthma patients and control group ($\overset{-}{x}\pm \mathrm{sd}$).

Group	*N*	NKT	CD4^+^NKT
Control	20	0.135 ± 0.061	63.28 ± 16.87
Asthma	42	0.051 ± 0.041^▲^	42.21 ± 13.72^▲^
SIT	24	0.091 ± 0.059^▲★^	50.45 ± 16.70^▲•^
*F*		18.594	12.698
*P*		0.000	0.000

Note: compared with control group, ^▲^ *P* < 0.01,

compared with asthma group, ^★^ *P* < 0.01, ^•^ *P* < 0.05.

**Table 3:** $\left(\overset{-}{x}\pm \mathrm{sd}\right)$.

Group	*N*	NKT	CD4^+^NKT
Intermittent	26	0.057 ± 0.045	44.21 ± 14.15
Mild persistent	10	0.046 ± 0.037	41.39 ± 11.39
Moderate persistent	6	0.035 ± 0.029	34.39 ± 14.99
*F*		0.757	1.148
*P*		0.476	0.328

**Table 4:** The level of IFN-*γ*, IL-4, IL-10 expression after NKT cells proliferation in asthma patients and control ($\overset{-}{x}\pm \mathrm{sd}$).

Group	IFN-*γ* (pg/mL)			IL-4 (pg/mL)			IL-10 (pg/mL)		
	24 H	48 H	72 H	24 H	48 H	72 H	24 H	48 H	72 H
Control (*n* = 15)	142.28 ± 54.31	410.11 ± 217.21	219.31 ± 96.12	11.96 ± 7.55	77.36 ± 37.42	93.05 ± 44.91	35.28 ± 18.27	100.71 ± 60.28	95.57 ± 60.90
Asthma (*n* = 20)	126.60 ± 40.63	374.79 ± 104.80	209.38 ± 96.61	28.71 ± 10.47^▲^	135.29 ± 40.90^▲^	277.60 ± 89.15^▲^	14.16 ± 5.29^▲^	28.60 ± 20.34^▲^	20.90 ± 11.50^▲^
SIT (*n* = 15)	149.92 ± 73.74	406.24 ± 211.08	226.82 ± 106.34	14.52 ± 8.68^★^	99.80 ± 48.25^★^	101.79 ± 57.90^★^	39.04 ± 28.65^★^	95.41 ± 49.62^★^	76.28 ± 32.36^★^
*F*	0.790	0.212	0.135	17.44	13.709	40.535	9.090	14.652	18.201
*P*	0.46	0.809	0.874	0.000	0.000	0.000	0.000	0.000	0.000

Note: compare with control group ^▲^ *P* < 0.01;

compare with asthma group ^★^ *P* < 0.01.

## Abbreviations

alpha-GalCer: Alpha-galactosylceramide
HDM: House dust mite
SIT: Specific immunotherapy
NKT: Natural killer T cells
TCR: T-cell receptor
iNKT: Invariant natural killer T cells
GSL: Glycosphingolipid.
